# Cardiac Specific Knockout of p53 Decreases ER Stress-Induced Mitochondrial Damage

**DOI:** 10.3389/fcvm.2019.00010

**Published:** 2019-02-19

**Authors:** Qun Chen, Jeremy Thompson, Ying Hu, Anindita Das, Edward J. Lesnefsky

**Affiliations:** ^1^Division of Cardiology, Departments of Medicine, Virginia Commonwealth University, Richmond, VA, United States; ^2^Biochemistry and Molecular Biology, Virginia Commonwealth University, Richmond, VA, United States; ^3^Physiology and Biophysics, Virginia Commonwealth University, Richmond, VA, United States; ^4^McGuire Department of Veterans Affairs Medical Center, Richmond, VA, United States

**Keywords:** mitochondria, complex I, thapsigargin, apoptosis, pyruvate dehydrogenase

## Abstract

Endoplasmic reticulum (ER) stress contributes to cardiovascular disease including heart failure. Interactions between the ER and mitochondria during ER stress can impair the mitochondrial respiratory chain and increase cell injury. p53 is a tumor suppressor protein that regulates apoptosis. p53 contributes to the regulation of mitochondrial and ER interactions, especially during the progression of ER stress. The knockout (KO) of p53 leads to decreased injury in hearts following ischemia-reperfusion. We asked if KO of p53 can protect mitochondria during the induction of ER stress and decrease cell injury. Floxed p53 mice were crossed with mice carrying an α-myosin heavy chain *cre* to generate cardiac specific p53 KO mice. Thapsigargin (THAP) was used to induce ER stress in wild type (WT) and p53 KO mice. Mice were euthanized after 48 h THAP treatment. Cardiac mitochondria were isolated for functional measurement. TUNEL staining was used to assess myocyte death. In WT mice, THAP treatment decreased the rate of oxidative phosphorylation using pyruvate + malate as complex I substrates compared to vehicle-treated control. Complex I activity was also decreased in the THAP-treated WT mice. The rate of oxidative phosphorylation and complex I activity were not altered in THAP-treated p53 KO mice. The content of pyruvate dehydrogenase (PDH) α1 subunit was decreased in THAP-treated WT mice but not in p53 KO mice. ER stress led to a release of cytochrome *c* and apoptosis inducing factor from mitochondria into cytosol in WT but not in KO mice. Knockout of p53 also preserved mitochondrial bcl-2 content in THAP-treated mice. In WT mice, THAP treatment markedly increased cell death compared to vehicle treated hearts. In contrast, cell injury was decreased in THAP-treated p53 KO mice compared to corresponding wild type. Thus, KO of p53 decreased cell injury by protecting mitochondria during the ER stress.

## Introduction

The endoplasmic reticulum (ER) contributes an essential role in protein synthesis and folding, lipid production, and calcium storage (1, 2). Many factors including calcium over load and oxidative stress lead to accumulation of unfolded proteins in the ER that results in a disturbance of the ER function known as ER stress (1, 2). The ER stress is involved in many cardiovascular diseases including heart failure and ischemia-reperfusion injury. Induction of ER stress by inhibiting Ca^2+^-ATPase with thapsigargin (THAP) increases cell injury in mouse hearts (3).

The ER and cardiac mitochondria are closely connected (4, 5) through mitochondria-associated membrane (MAM) domains (6). The MAM fraction provides direct physical contact between the ER and mitochondria. Formation of the contact sites between MAM and mitochondria is important to key cellular events including the transport of calcium from the ER to mitochondria and the formation of autophagosomes to regulate the morphology and cell survival (4, 6). Furthermore, p53 contributes to ER and mitochondrial interactions during ER stress (7). We proposed that ER mediated mitochondrial damage in cardiomyocytes may occur in part via the actions of p53.

Mitochondria are essential to support normal cardiac function via energy production and synthetic functions. Mitochondrial dysfunction is also a key source of increased cardiac injury (8–12). The ER stress is involved in mitochondrial dysfunction in rat hearts (2). THAP-induced ER stress leads to decreased oxidative phosphorylation, increased ROS (reactive oxygen species) generation, and sensitized MPTP (mitochondrial permeability transition pore) opening (2, 3). These results suggest that ER stress increases cell injury by inducing mitochondrial damage in mouse hearts. Complex I is the largest respiratory complex of the electron transport chain (ETC). Complex I is a key component to form the supercomplexes that enhance the efficiency of electron transport (13). Complex I is also a major source of ROS generation in cardiac mitochondria (14). Chronic inhibition of complex I activity facilitates the development of heart failure (15). Thus, we asked if ER stress led to decreased complex I-mediated respiratory and enzyme activity.

In addition to complex I, inhibition of the pyruvate dehydrogenase (PDH) complex also contributes to the development of heart failure (16, 17). Fatty acids and carbohydrates are two main substrate classes that generate ATP to support cardiac function. In the resting state, the majority of ATP (> 70%) is generated from fatty acid oxidation. The remaining ATP comes from the oxidation of pyruvate produced from glucose. Under increased workload conditions, the generation of ATP from carbohydrate is markedly increased to meet the high-energy demand of the heart (18). Therefore, it is important to maintain substrate flexibility (a balance between glucose usage and fatty acid metabolism) to support normal cardiac function. PDH is a rate-limiting enzyme to catalyze pyruvate oxidation to acetyl-CoA that enters the tricarboxylic acid (TCA) cycle (19, 20). Inhibition of PDH impairs substrate flexibility and energy generation, especially in the setting of increased cardiac workload. Thus, prolonged PDH inhibition can contribute to the development of heart failure (18). Ischemia-reperfusion leads to degradation of PDH through activation of the mitochondrial matrix localized calpain 1 that is a calcium sensitive cysteine protease (21). ER stress increases calcium over load by disturbing calcium storage (2). We propose that the ER stress can lead to PDH degradation as a further mechanism of impaired mitochondrial function.

p53 is a tumor suppressor protein that regulates cell cycle and DNA repair pathways (22, 23). p53 is also a key regulator of apoptosis during multiple pathological conditions. p53 increases apoptosis by acting as a transcription factor to increase or decrease pro-apoptotic and anti-apoptotic target gene expression (23, 24). p53 can also increase apoptosis by causing direct release of cytochrome c from mitochondria through permeation of the outer mitochondria membrane via interaction with bcl-2 family proteins (25). Knockout of p53 decreases the release of cytochrome c from mitochondria into cytosol during cardiac toxicity (26). Induction of ER stress in mouse embryonic fibroblasts increases apoptosis via the enhanced expression of BH3-only proteins including PUMA (p53-up-regulated modulator of apoptosis) and NOXA (Phorbol-12-myristate-13-acetate-induced protein 1). Knockout of p53 in mouse embryonic fibroblasts decreases apoptosis during ER stress by inhibiting PUMA and NOXA expression. These results indicate that p53 is involved in the ER stress-induced apoptosis (27–29). p53 localizes to the ER and affects mitochondrial-ER interactions during ER stress (7). However, it is unclear if p53 contributes to mitochondrial damage during the ER stress. In the current study, we studied the role of p53 in ER stress-mediated injury to cardiac mitochondria.

## Methods

### Induction of the ER Stress in C57BL/6 Mice

The Animal Care and Use Committee of the McGuire VA Medical Center approved the study. Floxed p53 mice in c57bl/6 background were crossed with mice carrying α-myosin heavy chain *cre* to generate cardiac specific p53 knockout (cardiac-specific KO) mice. Both floxed p53 mice and α-myosin heavy chain *cre* mice were purchased from Jackson Laboratory (Bar Harbor, Maine). Primers used for genotype PCR assay are: Cre-1: GCG GTC TGG CAG TAA AAA CTA TC; Cre-2: GTG AAA CAG CAT TGC TGT CAC TT. p53-1: GGT TAA ACC CAG CTT GAC CA; p53-2: GGA GGC AGA GAC AGT TGG AG. Mice were in the C57BL/6 background and 2–3 month old mice were used in the current study. Mice received a normal diet with *ad libitum* access to food and water during the experiment. THAP (3 mg/kg) was dissolved in DMSO and diluted with saline to induce ER stress through one-time i.p. injection in mice without fasting (2). Control mice received vehicle (DMSO) treatment. Mice were anesthetized with pentobarbital sodium (90 mg/kg, i.p.) 48 h after one-time THAP treatment (3). The mouse heart was quickly excised for mitochondrial isolation or histological examination.

### Determination of Apoptotic Cell Death

Apoptotic cell death in myocardium was analyzed by TUNEL staining, using a commercial kit (BD Biosciences, San Jose, CA) that detects nuclear DNA fragmentation via fluorescence assay. In brief, mouse hearts from wild type or knockout with or without THAP treatment were excised and stored in a 10% formalin solution. Myocardium apoptosis was detected using ApopAlert DNA Fragmentation Assay Kit purchased from BD Biosciences (San Jose, CA) that detects nuclear DNA fragmentation. The assay is based on terminal deoxynucleotidyl transferase (TdT)-mediated incorporation of fluorescein-dUTP at the free 3'-hydroxyl ends of the fragmented DNA. In brief, formalin-fixed, paraffin-embedded tissue sections was mounted on glass slides. After de-paraffinized the slides with xylene and ethanol, slides were microwaved for 10 min with Citrate Buffer (pH 6.0). After washing with PBS (phosphate-buffered saline, pH 7.4), slides were incubated with TUNEL staining according to the manufacture's protocol. The slides were then counterstained with Vectashield mounting medium with 4′, 6-diamidino-2-phenylindole (DAPI, Vector Laboratories). The fluorescein-labeled DNA and all nuclei with DAPI were quantified using fluorescence microscopy. Apoptosis was assessed in transverse paraffin sections with TUNEL staining (30). The apoptotic index was expressed as the number of apoptotic cells of all cardiomyocytes per field. The apoptotic rate was calculated using 10 random fields per slide. The transverse sections were then counterstained with Vectashield mounting medium with 4,6-diamidino-2-phenylindole (a DNA intercalating dye for visualizing nuclei in fixed cells; catalog number H-1200, Vector Laboratories, Burlingame, CA). The stained cells were examined under an Olympus IX70 fluorescence microscope (31).

A small piece of myocardium was fixed for electron microcopy analysis of mitochondrial morphology (magnification 100 KX). Myocardial samples were immersed into 3% buffered glutaraldehyde. The myocardium tissue was processed into resin and cut for transmission electron microscopy (32).

### Isolation of Cytosol and Mitochondria

Heart mitochondria were isolated as previously described (33). The mouse heart was placed in cold buffer A (composition in mM: 100 KCl, 50 MOPS [3–(N–morpholino) propanesulfonic acid], 1 EGTA, 5 MgSO_4_, and 1 mM ATP]. The heart was blotted dry, weighed, and homogenized using a polytron tissue homogenizer at 10,000 rpm for 2.5 s with trypsin (5 mg/g tissue). Trypsin was used to generate a combined population of cardiac mitochondria from a single mouse heart. Trypsin treatment also removed potential cytosolic contamination. The homogenate was incubated for 15 min at 4°C, then the same volume of buffer B [buffer A + 0.2% bovine serum albumin (BSA)] was added and the mixture was centrifuged at 500 × g for 10 min. The supernatant was again centrifuged at 3,000 × g to pellet mitochondria. The mitochondrial pellet was first washed with buffer B, then re-suspended in KME (100 mM KCl, 50 mM MOPS, 0.5 mM EGTA), and centrifuged at 3,000 × g to yield the final mitochondrial pellet. Mitochondria were re-suspended in KME for study (33).

Oxygen consumption in mitochondria was measured using a Clark-type oxygen electrode at 30°C as previously described (33). Pyruvate (20 mM) + Malate (10 mM) were used as complex I substrate. Succinate (20 mM, complex II substrate) was used as the complex II substrate with the inclusion of 7.5 μM rotenone. ADP (2 mM) was used to determine the maximal rate of ADP-stimulated respiration (34).

### Western Blotting

Proteins were separated using 12 or 4–15% Tris-glycine gels (Bio-Rad, Hercules, CA) and transferred to PVDF membrane (Millipore) using semi-dry transfer (Bio-Rad). The blots were incubated for 1 h at room temperature in 5% (w/v) non-fat dry milk (Bio-Rad) in TBST buffer (10 mM Tris pH 7.5, 150 mM NaCl, 0.1% Tween20) followed by the overnight incubation at 4°C with primary antibody. After 1 h incubation at room temperature with a 1:10,000 dilution of HRP-conjugated anti-mouse or anti-rabbit IgG F(ab)_2_ (GE Healthcare Life Sciences, Piscataway, NJ), blots were developed using ECL Plus Western Blotting Detection Reagents (GE Healthcare Life Sciences, Piscataway, NJ) (3). The protein bands were quantitated with ChemiDoc Imager and Image Lab^TM^ software (Bio-Rad, Hercules, CA).

### Statistical Analysis

Data are expressed as the mean ± standard error (35). For all analyses, differences between groups in biologic variables and mitochondrial function were compared by one-way ANOVA. When a significant *F*-value was obtained, means were compared using the Student-Newman-Keuls test of multiple comparisons. Statistical significance was defined as a value of *p* < 0.05.

## Results

### Knockout of p53 Decreases Cell Death During ER Stress

**Figure 1:**
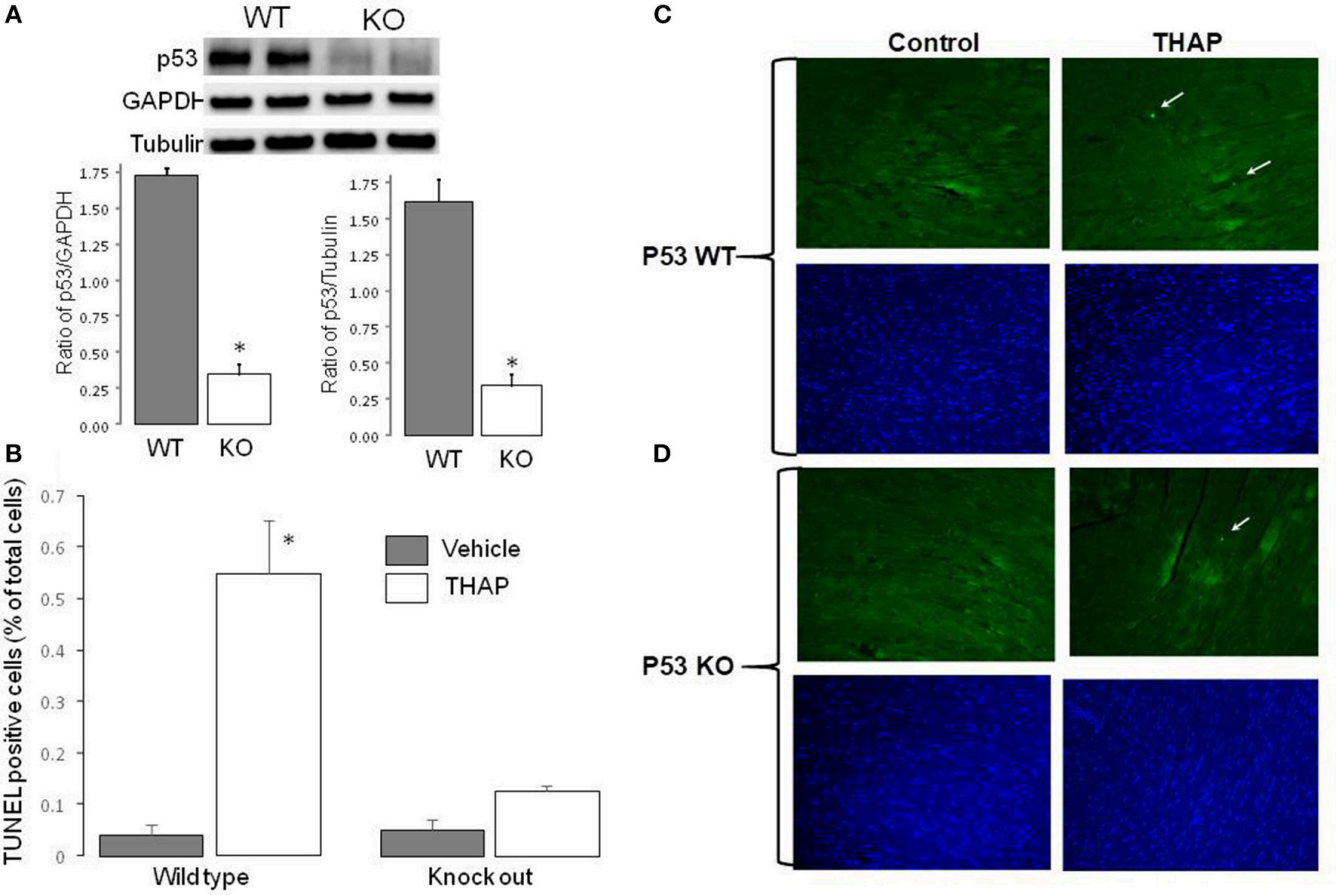
Knockout of p53 decreases cardiac injury in THAP-treated mouse hearts. The content of p53 in knockout mouse heart homogenate was markedly deceased compared to wild type **(A)**. GAPDH and Tubulin were used as protein loading control **(A)**. THAP (3 mg/kg) was administrated through intraperitoneal injection to induce the ER stress for 48 h in wild type and p53 knockout mice. Apoptotic cell death was assessed using TUNEL staining [green color, **(C,D)**]. Total nuclei were quantified using DAPI staining [blue color, **(C,D)**]. Arrows indicated a typical TUNEL positive nucleus. THAP significantly increased apoptotic cell death in wild type, but not in p53 knockout mice **(B)**. Mean ± SEM. ^*^p < 0.05 vs. vehicle. *n* = 3 in each group.

The content of p53 was markedly decreased in cardiac-specific KO mice compared to wild type (WT) (Figure 1A). GAPDH and tubulin were used as protein loading control. There were no differences in the contents of GAPDH and tubulin between WT and cardiac-specific KO mice (Figure 1A). Therefore, GAPDH was used as protein loading control in the current study. TUNEL staining was used to assess apoptosis in mouse hearts with THAP treatment. Compared to vehicle-treated WT control, the frequency of apoptotic nuclei was significantly increased in THAP treated hearts (Figure 1B), indicating that the THAP-induced ER stress increased apoptotic cell death. However, knockout of p53 markedly decreased the frequency of apoptotic nuclei observed in THAP-treated hearts (Figure 1B), supporting that knockout of p53 decreases programmed cell death during ER stress. Representative pictures of TUNEL staining were shown in Figure 1C (wild type) and Figure 1D (knockout mice).

**Figure 2:**
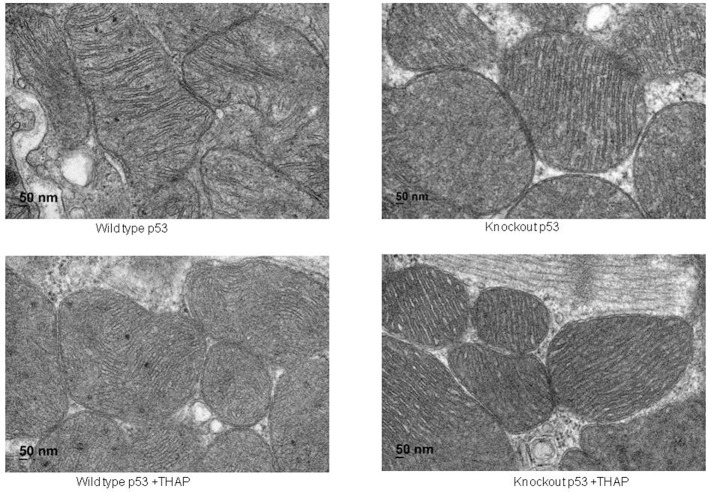
Electron micrograph of cardiac mitochondria in a portion of myocyte. Mitochondria were well-preserved and relatively intact in mouse hearts with or without THAP treatment. Knockout of p53 did not lead to significant alteration of mitochondrial morphology. Magnification x 100 K.

Electron microcopy study showed that there were no significant alterations in mitochondrial morphology between WT and cardiac-specific KO mice (Figure 2). THAP treatment also did not markedly alter the morphology in myocardium from either WT and cardiac-specific KO mice (Figure 2).

### THAP Treatment Increased the ER Stress in Both Wild Type and Knockout Mice

**Figure 3:**
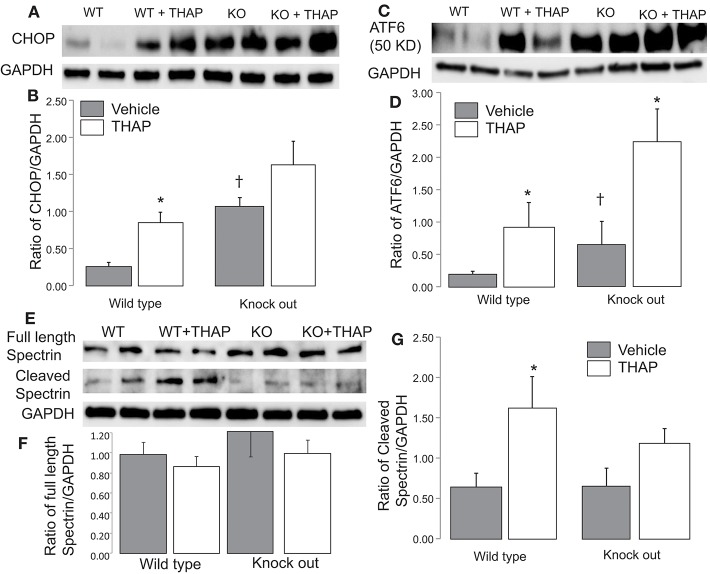
Administration of THAP increases the ER stress in both wild type and knockout mice THAP treatment markedly increased cytosolic CHOP content in wild type compared to vehicle control. THAP trended to increase CHOP in p53 knockout mice **(A,B)**. THAP increased the content of cleaved ATF6 in both wild type and p53 knockout mice **(C,D)**. These results support that THAP increase the ER stress in both wild type and knockout mice. THAP treatment did not significantly alter the full length of spectrin in both wile type and p53 knockout mice **(E,F)**. THAP treatment increases the cleavage of spectrin, a marker of cytosolic calpain activation, whereas knockout of p53 attenuated the spectrin cleavage **(E,G)**, suggesting that knockout of p53 attenuates calpain activation during the ER stress. GAPDH was used as protein loading control in each group. Mean ± SEM. *p < 0.05 vs. vehicle. ^†^*p* < 0.05 vs. corresponding wild type vehicle. *n* = 4 in each group.

CHOP (C/EBP homologous protein) expression and cleaved ATF 6 (50 Kd) are commonly used as a downstream mediator of ER stress induced cell injury (36). THAP treatment significantly increased the CHOP content in WT mice, supporting that THAP treatment increases the ER stress (Figures 3A,B). THAP tended to increase CHOP expression in cardiac-specific KO mice (Figures 3A,B). THAP treatment increased the cleavage of ATF6 in both WT and cardiac-specific KO mice compared to vehicle treatment (Figures 3C,D). These results support that THAP increased the ER stress in both WT and cardiac-specific KO mice. Interestingly, the contents of CHOP and cleaved ATF6 were higher in cardiac-specific KO mice than that in WT mice in basal condition (Figures 3C,D), suggesting that knockout of p53 alone increases the ER stress.

ER stress leads to calcium over load that activates calpains. An increase in cleaved spectrin content is an indicator of the cytosolic calpain activation (37). In WT mice, THAP treatment trended to decrease the content of full length of spectrin, but it did not reach the statistical difference (Figures 3E,F). There were no differences in full length spectrin content in p53 cardiac-specific KO mice with or without THAP treatment (Figures 3E,F). In wild type mice, THAP treatment increases the cleaved spectrin content compared to vehicle (Figures 3E,G), supporting the ER stress activates the cytosolic calpains. THAP treatment did not increase the cleaved spectrin in p53 knockout mice (Figures 3E,G), suggesting that knockout of p53 attenuates cytosolic calpain activation during the ER stress.

### Knockout of p53 Protected Complex I and PDH During ER Stress

**Figure 4:**
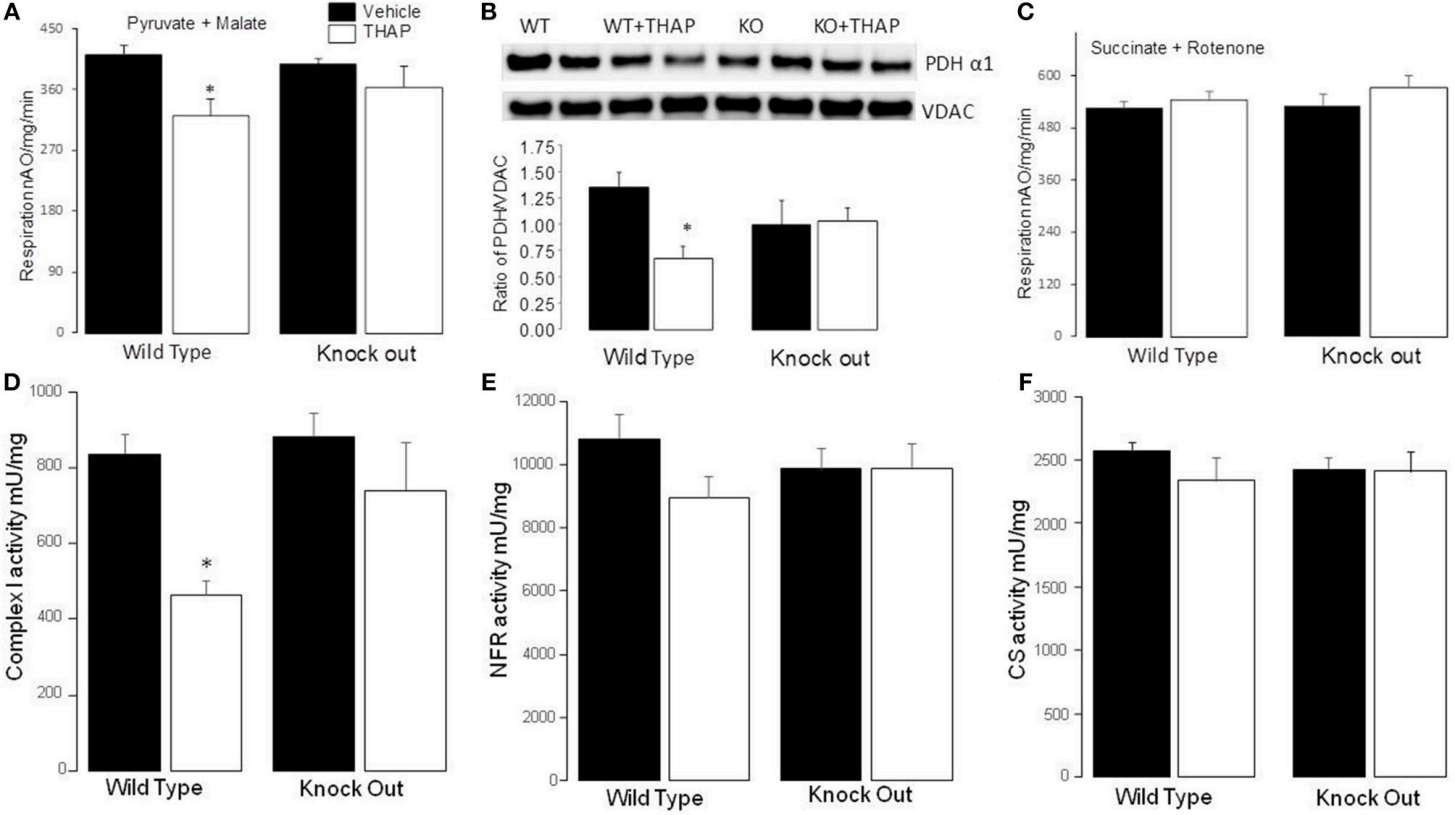
Knockout of p53 improves oxidative phosphorylation and complex I activity during the ER stress. Compared to control, THAP treatment decreased the rate of oxidative phosphorylation in wild type mice when pyruvate + malate was used as complex I substrate **(A)**. The rate of oxidative phosphorylation was maintained in p53 knockout mice with THAP treatment **(A)**. THAP treatment led to degradation of PDHα1 subunit in wild type mice but not in p53 knockout mice **(B)**. VDAC was used as protein loading control. There were no differences in the oxidation of succinate (+rotenone) between groups **(C)**. THAP also decreased complex I activity in wild type mouse heart mitochondria. The complex I activity was protected in p53 knockout mice during THAP treatment **(D)**. THAP treatment did not alter the activities of NFR **(E)**, and citrate synthase (CS) **(F)** in both wild type and knockout mice. Mean ± SEM; *p < 0.05 vs. vehicle. *N* = 4–8 in each group.

In WT mice, THAP treatment led to decreased complex I activity compared to vehicle (Figure 4A). The complex I activity was not altered in p53 cardiac-specific KO mice with THAP treatment (Figure 4A). NADH dehydrogenase activity (NFR) (Figure 3B) and citrate synthase (CS) (Figure 3C) were not altered with THAP treatment in both WT and p53 cardiac-specific KO mice. NFR is the first part of complex I (38). THAP treatment decreases complex I activity without alteration in NFR activity, indicating that the damage site of THAP treatment is distal to the NADH dehydrogenase portion of the complex. The rate of oxidative phosphorylation was also measured using mitochondria isolated from mouse hearts with or without THAP treatment. THAP led to decreased rate of state 3 and increased rate of state 4 respiration in WT but not in p53 cardiac-specific KO mice when pyruvate + malate were used as complex I substrates (Table 1). The respiratory control ratio (RCR) was also decreased in THAP-treated WT with complex I substrates. These results indicated that THAP treatment not only led to damage in the electron transport chain, but also impaired the inner mitochondrial membrane intactness as shown the increased state 4 respiration. THAP treatment did not alter the state 3 and state 4 respiration in both WT and cardiac-specific KO mice when succinate was used as complex II substrates (Table 1). In wild type mice, THAP treatment decreased the rate of high-ADP stimulated oxidative phosphorylation when pyruvate + malate were used as complex I substrates (Figure 4D). THAP treatment did not alter pyruvate oxidation in p53 cardiac-specific KO mice (Figure 4D). Immunoblotting showed that THAP treatment led to decreased protein content of PDH α1 subunit in WT but not in p53 cardiac-specific KO mice (Figure 4E). THAP did not alter the oxidative phosphorylation using succinate as the complex II substrate (rotenone was used to block potential reverse electron flow from complex II to complex I) in both WT and cardiac-specific KO mice (Figure 4F).

**Table 1:** ER stress decreased the ADP-stimulated respiration in cardiac mitochondria using pyruvate + malate as substrates.

	**WT (*n =* 12)**	**WT + THAP (*n =* 7)**	**KO (*n =* 10)**	**KO + THAP (*n =* 6)**
**COMPLEX I SUBSTRATES: PYRUVATE** **+** **MALATE**				
State 3	370 ± 10	284 ± 21^*^	350 ± 10	348 ± 30
State 4	80 ± 5	102 ± 6^*^	93 ± 5	111 ± 10
RCR	4.4 ± 0.1	2.8 ± 0.2^*^	3.8 ± 0.2	3.2 ± 0.1
**COMPLEX II SUBSTRATES: SUCCINATE**				
State 3	579 ± 21	587 ± 19	598 ± 21	610 ± 24
State 4	173 ± 3	179 ± 8	193 ± 10	185 ± 11
RCR	3.3 ± 0.1	3.3 ± 0.1	3.2 ± 0.2	3.3 ± 0.1

Mean ± SEM:

* *p < 0.05 vs. WT*.

### Knockout of p53 Decreased Cytochrome c Loss During ER Stress

**Figure 5:**
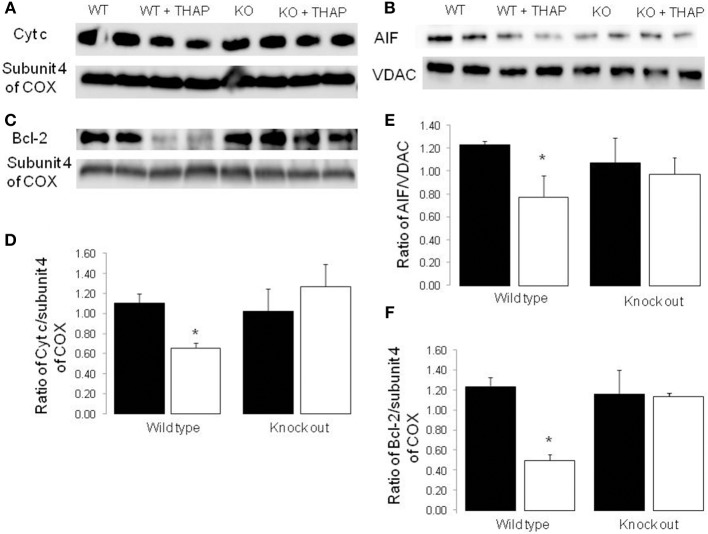
Knockout of p53 decreases the loss of cytochrome *c* and AIF from mitochondria during the ER stress Immunoblotting showed that THAP treatment led to decreased contents of cytochrome *c* and AIF in wild type mice, but not in p53 knockout mice **(A,B,D,E)**. THAP treatment also markedly decreased bcl-2 content in wild type mice **(C,F)**. The attenuation of bcl-2 content was decreased in THAP-treated p53 knockout mice **(C,F)**. These results suggest that knockout of p53 decreases the loss of cytochrome *c* and AIF through attenuation of the outer membrane permeability during the ER stress. VDAC was used as protein loading control for AIF. Subunit 4 of complex IV was used as protein loading control for cytochrome *c* and bcl-2. Mean ± SEM. *p < 0.05 vs. vehicle. *n* = 4 in each group.

THAP TREATMENT significantly decreased the content of cytochrome *c* in mitochondria isolated from WT mice compared to vehicle control (Figures 5A,D). The content of cytochrome *c* was not altered in p53 cardiac-specific KO mice with THAP treatment. Subunit 4 of cytochrome oxidase was used as a loading control for the cytochrome *c* (Figures 5A,D). In addition, THAP treatment also decreased the content of AIF (apoptosis inducing factor) in WT mouse heart mitochondria (Figures 5B,E). THAP did not lead to decreased AIF content in p53 cardiac-specific KO mice (Figures 5B,E). Bcl-2 content was markedly decreased in WT mouse heart mitochondria with THAP treatment (Figures 5C,F). THAP treatment did not alter the bcl-2 content in p53 cardiac-specific KO mice (Figures 5C,F).

## Discussion

In the present study, we show that knockout of p53 decreases cell death from THAP-induced ER stress in mouse hearts. Knockout of p53 decreases mitochondrial damage from the ER stress as shown by improved oxidative phosphorylation, protected complex I activity, and PDH content and retention of cytochrome *c* and AIF from mitochondria in THAP-treated mice. Knockout of p53 also improves bcl-2 content in cardiac mitochondria isolated from THAP-treated hearts. These results indicate that knockout of p53 decreases the ER stress-induced cell death at least in part by protecting cardiac mitochondria.

Mitochondrial electron transport chain damage contributes to cardiac injury during stress conditions (8, 9). Although acute and brief complex I inhibition can decrease cardiac injury during ischemia-reperfusion (39), prolonged, chronic complex I inhibition is detrimental to cardiomyocytes (15, 40). The decreased complex I activity in THAP treated mice supports that ER stress contributes to complex I damage. Inhibition of complex I through inactivation of the gene for the nuclear-encoded complex I subunit Ndufs4 facilitates the development of heart failure following pressure overload (15). Inhibition of complex I through knockdown of the gene for the complex I subunit Ndufs6 leads to decreased ATP production and heart failure as well (41). These results support that chronic complex I inhibition contributes to the development of heart failure. The decreased complex I activity in THAP treated mice supports that ER stress contributes to complex I damage. Knockout of p53 protects complex I activity in THAP-treated mice. These results suggest that the protection from downregulation of p53 is mediated by maintaining complex I activity. In addition to complex I, knockout of p53 also protects PDH during the ER stress. In the current study, we show that ER stress leads to decreased pyruvate oxidation accompanied by degradation of the PDH α1 subunit. Knockout of p53 improves oxidative phosphorylation in THAP-treated mice with pyruvate as complex I substrate with concomitant preservation of the content of the PDH α1 subunit.

PDH is a critical enzyme complex with regard to metabolic substrate flexibility in the heart (42). In order to meet the constant high energy demand, it is critical to maintain cardiac metabolic flexibility that enables the heart to orchestrate ATP production using divergent energy substrates including fatty acids and carbohydrates during different conditions to maintain contractile function (17). The impaired metabolic flexibility contributes to heart failure development (43). Genetic knockout of the PDHα1 subunit increases cardiac injury during ischemia (44). Thus, knockout of p53 can decrease cardiac injury through protection of PDH leading to preserved oxidative phosphorylation with pyruvate as substrate.

In buffer-perfused mouse hearts, ischemia-reperfusion dramatically decreased the PDH α1 subunit content (21). Administration of the calpain inhibitor (MDL-28170) preserves PDH content in mouse heart mitochondria following ischemia-reperfusion (21), suggesting that activation of mitochondrial calpains contributes to PDH damage. Incubation of calcium with purified heart mitochondria leads to PDH degradation, whereas MDL-28170 treatment protects PDH in calcium-treated mitochondria. Genetic inhibition of mitochondrial calpain 1 activity prevents calcium-mediated PDH degradation (Chen and Lesnefsky, unpublished data). These results support that activation of mit-CPN1 in the mitochondrial matrix contributes to PDH damage.

AIF is a substrate of mitochondrial calpain 1 that is located in the mitochondrial inter-membrane space and the matrix (45). AIF is bound on the inner mitochondrial membrane. Activation of mitochondrial calpain 1 is required to detach AIF from the inner membrane by cleaving AIF to truncated AIF that is released into cytosol (45). Calpain-mediated cleavage of AIF in the mitochondrial intermembrane space is the initial step leading to AIF release (23, 24). ER stress increases the cleavage of cytosolic spectrin, providing direct evidence of calpain activation (46). THAP increases ER stress by inducing intracellular Ca^2+^ overload through inhibition of Ca^2+^-ATPase in the ER (3). Calpain 1 is a calcium-dependent protease. Intracellular calcium overload during the ER stress leads to calpain 1 activation and subsequent enzyme damage (45). Thus, our results suggest that THAP-induced ER stress likely leads to decreased PDH content by activating mitochondrial calpain 1. Knockout of p53 preserves the PDH content, suggesting that knockout of p53 prevents mitochondrial calpain activation during the ER stress. The role of p53 in ER-mitochondrial interactions to regulate calcium loading of mitochondria is in line with this notion (7). Knockout of p53 is expected to decrease calcium loading of mitochondria, in turn likely to attenuate activation of mitochondrial calpain, with reduced AIF cleavage and release as well as preservation of PDH α1 content.

A release of cytochrome *c* into cytosol increases caspase-dependent apoptosis, whereas a release of AIF into cytosol increases caspase-independent apoptosis (47). THAP treatment leads to release of both cytochrome *c* and AIF into cytosol, indicating that the ER stress increases both caspase-dependent and caspase-independent apoptosis. Knockout of p53 decreases cell death by preventing the loss of cytochrome *c* and AIF from mitochondria. The cytochrome *c* and AIF are present in the mitochondrial intermembrane space. Permeation of the outer mitochondrial membrane is required for them to be released into cytosol, providing the second step required for AIF release following cleavage to truncated AIF following calpain activation. A decrease in bcl-2/bax ratio can also increase outer membrane permeability (48, 49). In the current study, bcl-2 content is markedly decreased in wild type hearts following THAP treatment. Bax content is not altered in mitochondria by THAP treatment (data not shown). Knockout of p53 maintains bcl-2 content following THAP treatment. These results suggest that knockout of p53 protects cytochrome *c* and AIF by preserving bcl-2 content during the ER stress. Knockout of p53 decreases the expression of PUMA and NOXA in MEFs during the ER stress. PUMA and NOXA increases cell death through the mitochondrial pathway of apoptosis. Thus, knockout of p53 may also decrease cytochrome *c* release into cytosol through inhibition of PUMA and NOXA expression (50). A recent study showed that knockout of p53 can decrease cocaine-induced apoptosis by disrupting the interaction between p53 and PKCδ kinase. Activation of PKCδ kinase led to increased cytochrome *c* content and caspase 3 activation in cytosol by decreasing bcl-2 and bcl-xl contents. Thus, p53-mediated PKCδ kinase activation may be a potential mechanism of bcl-2 depletion during the ER stress (26).

Although the initial response to ER stress is to restore ER function by activating specific signaling pathways to increase protein chaperone expression, severe ER stress inevitably increases cell injury and favors cell death (1, 51). In the current study, THAP treatment increases cytosolic CHOP expression. An increase in CHOP content induces apoptosis during the ER stress. However, contradictory results also exist. Knockout of CHOP alone is not sufficient to induce apoptosis (52). In the current study, CHOP content is higher in p53 knockout mice compared to wild type. In addition, knockout of p53 does not decrease CHOP expression. These results were consistent with the previous study that knockout of p53 did not alter the CHOP expression in the mouse embryonic fibroblasts during ER stress (27). The current study highlights that knockout of p53 protects cells through prevention of the ER stress-mediated mitochondrial damage, rather than direct inhibition of the ER stress.

There are limitations to the current study. Since trypsin is used to isolate mitochondria, we cannot detect p53 translocation to mitochondria during the ER stress in that potential translocated p53 may be removed during the trypsin treatment. Percoll purification may be used in the future to study if the THAP-induced ER stress leads to p53 translocation to mitochondria. A high-resolution immunofluorescence confocal microscopy is another tool to study the effect of ER stress on p53 translocation to mitochondria in myocytes from wild type and p53 knockout cells. In addition, cardiac function was not measured in the current study due to technical limitation. A deterioration of cardiac function in THAP-treated wild type but not knockout hearts would provide further evidence to support our conclusions.

Knockout of p53 decreases cardiac injury during ischemia-reperfusion and post-ischemic ventricular remodeling (53). The current study shows that knockout of p53 can decrease cell injury during the ER stress mediated via protection of mitochondria from ER-induced damage. ER stress occurs in aging (54) and heart failure (55). Thus, modulation of cardiac p53 expression may be a strategic approach to decrease cell injury in the settings that lead to cardiac injury and eventual heart failure.

## Author Contributions

QC: experimental design, performing experiment, data analysis, manuscript preparation, and writing. JT: performing experiment; YH: performing experiment; AD: performing experiment and data analysis; EL: experimental design, manuscript editing.

### Conflict of Interest Statement

The authors declare that the research was conducted in the absence of any commercial or financial relationships that could be construed as a potential conflict of interest.
